# Substitute parameters of exercise-induced pulmonary hypertension and usefulness of low workload exercise stress echocardiography in mitral regurgitation

**DOI:** 10.1038/s41598-022-19987-8

**Published:** 2022-09-25

**Authors:** Masashi Amano, Shoko Nakagawa, Kenji Moriuchi, Hitomi Nishimura, Yurie Tamai, Ayaka Mizumoto, Yoshiki Yanagi, Rika Yonezawa, Yutaka Demura, Yoshito Jo, Yuki Irie, Atsushi Okada, Takeshi Kitai, Makoto Amaki, Hideaki Kanzaki, Kengo Kusano, Teruo Noguchi, Kunihiro Nishimura, Chisato Izumi

**Affiliations:** 1 Department of Cardiovascular Medicine, National Cerebral and Cardiovascular Center, 6-1 Kishibe-shimmachi, Suita, Osaka 564-8565 Japan; 2 Department of Clinical Laboratory, National Cerebral and Cardiovascular Center, Suita, Japan; 3 Department of Preventive Medicine and Epidemiology, National Cerebral and Cardiovascular Center, Osaka, Japan

**Keywords:** Cardiology, Outcomes research

## Abstract

In asymptomatic patients with mitral regurgitation (MR), data of exercise-induced pulmonary hypertension (EIPH) are limited, and feasibility of evaluating EIPH is not high. We aimed to investigate prognostic impact of EIPH and its substitute parameters. Exercise stress echocardiography (ESE) were performed in 123 consecutive patients with moderate to severe degenerative MR. The endpoint was a composite of death, hospitalization for heart failure, and worsening of symptoms. EIPH [tricuspid regurgitation peak gradient (TRPG) at peak workload ≥ 50 mmHg] was shown in 57 patients (46%). TRPG at low workload was independently associated with TRPG at peak workload (β = 0.67, p < 0.001). Early surgical intervention (within 6 months after ESE) was performed in 65 patients. Of the remaining 58 patients with the watchful waiting strategy, the event free survival was lower in patients with EIPH than in patients without EIPH (48.1 vs. 97.0% at 1-year, p < 0.001). TRPG at low workload ≥ 35.0 mmHg as well as EIPH were associated with poor prognosis in patients with the watchful waiting strategy. In conclusion, the importance of ESE and evaluating EIPH in patients with MR was re-acknowledged. TRPG at peak workload can be predicted by TRPG at low workload, and TRPG at low workload may be useful in real-world clinical settings.

## Introduction

Degenerative severe mitral regurgitation (MR) induces elevation of left atrial (LA) pressure and leads to pulmonary hypertension (PH) before the development of symptoms or left ventricular (LV) dysfunction^1^. In the current guidelines, surgical intervention for MR is indicated in patients with resting PH [resting systolic pulmonary artery pressure (SPAP) > 50 mmHg] as well as being symptomatic, or LV dysfunction^2,3^. Conversely, in asymptomatic patients with severe MR without resting PH, exercise stress echocardiography (ESE) has been recommended in the guidelines as an additional test in decision-making for surgical intervention to unmask the symptoms^2–4^ or to demonstrate PH during exercise^2,3^ because the exercise-induced PH (EIPH) (exercise SPAP > 60 mmHg at peak workload) was a predictor of low survival and occurrence of symptoms during follow-up^5,6^. Moreover, in patients with moderate MR with equivocal symptoms, the ESE is also reasonable because exercise induced severe MR is concealed at rest and exertional symptoms or EIPH is often evoked^7^. However, data are limited on how the results of ESE and EIPH affect prognosis in patients with degenerative MR in real-world clinical settings^2–4^.

There are several problems in ESE. First, at peak workload, it is difficult to accurately quantify tricuspid regurgitation velocity for estimating SPAP due to poor image under tachycardia and tachypnea^8^. Second, poor exercise performance due to muscle weakness is also problem, especially in elderly patients^9^. Therefore, if EIPH at peak workload is predicted by data at low workload, the usefulness of performing exercise stress test for patients with MR would increase.

This study aimed to investigate (1) prognostic impact of EIPH in asymptomatic patients with moderate to severe MR and (2) substitute parameters of EIPH at peak workload.

## Results

### Patients’ characteristics

The overall baseline characteristics are shown in Table 1. Patients were divided based on the occurrence of EIPH into 2 groups: patients with EIPH at peak workload (TRPG ≥ 50 mmHg, EIPH group, n = 57) and patients without EIPH at peak workload (TRPG < 50 mmHg, no-EIPH group, n = 66). The baseline characteristics according to the occurrence of EIPH are also shown in Table 1. The EIPH group was older (70.4 ± 10.7 vs. 61.1 ± 17.3 years, p < 0.001), and had a larger percentage of NYHA class II (54% vs. 33%, p = 0.019) than the no-EIPH group. Other background and laboratory data, including renal function and brain natriuretic peptide level, were similar between the 2 groups.

**Table 1:** Baseline characteristics.

	Overall (N = 123)	EIPH (N = 57)	No-EIPH (N = 66)	p-value
Age, years old	65.4 ± 15.3	70.4 ± 10.7	61.1 ± 17.3	< 0.001
< 50 years old, n (%)	20 (16)	3 (5)	17 (26)	0.002
Sex (male), n (%)	68 (55)	34 (60)	34 (52)	0.37
Body surface area, m^2^	1.61 ± 0.19	1.63 ± 0.17	1.60 ± 0.20	0.34
Atrial fibrillation, n (%)	18 (15)	8 (14)	10 (15)	0.86
NYHA II, n (%)	53 (43)	31 (54)	22 (33)	0.019
Hypertension, n (%)	58 (47)	32 (56)	26 (39)	0.064
Diabetes, n (%)	6 (4.9)	1 (1.8)	5 (7.6)	0.14
Current or previous smoking, n (%)	27 (22)	16 (28)	11 (17)	0.13
Hyper lipidemia, n (%)	36 (29)	21 (37)	15 (23)	0.086
Previous PCI, n (%)	2 (1.6)	1 (1.8)	1 (1.5)	0.92
Previous cerebral infarction, n (%)	1 (0.8)	1 (1.8)	0	0.28
Hemoglobin, g/dL	13.6 ± 1.4	13.4 ± 1.4	13.8 ± 1.3	0.21
Creatinine, mg/dL	0.86 ± 0.25	0.86 ± 0.24	0.86 ± 0.25	0.98
Estimated glomerular filtration rate, mL/min/1.73m^2^	65.3 ± 16.6	62.9 ± 14.1	67.4 ± 18.4	0.14
Brain natriuretic peptide, pg/mL	42.7 (20.1–96.3)	63.2 (35.0–136.1)	27.6 (15.3–81.2)	0.097

*EIPH* exercise-induced pulmonary hypertension, *NYHA* New York heart association, *PCI* percutaneous coronary intervention.

### Comparison of symptoms and vital signs at peak workload between EIPH and no-EIPH group

There were no differences in workload at peak effort (79.0 ± 25.4 vs. 85.2 ± 29.1 W, p = 0.21), values of the Borg scale related to dyspnea (16.0 ± 2.2 vs. 16.1 ± 2.4, p = 0.80) and leg fatigue (15.8 ± 2.7 vs. 16.5 ± 2.4, p = 0.13), and vital signs at peak workload between the EIPH and no-EIPH groups (Table 2).

**Table 2:** Hemodynamics at peak workload.

	Overall (N = 123)	EIPH (N = 57)	No-EIPH (N = 66)	p-value
Systolic blood pressure, mmHg	181.1 ± 25.5	182.3 ± 24.7	180.2 ± 26.3	0.65
Diastolic blood pressure, mmHg	93.2 ± 18.3	91.7 ± 18.8	94.6 ± 17.8	0.38
Heart rate, bpm	128.9 ± 18.7	125.5 ± 17.0	131.9 ± 19.7	0.06
Stroke volume, mL	66.1 ± 15.8	67.2 ± 14.6	65.1 ± 16.9	0.46
Cardiac index, L/min/m^2^	5.25 ± 1.31	5.18 ± 1.25	5.30 ± 1.36	0.62
Tricuspid regurgitation pressure gradient, mmHg	48.6 ± 11.6	58.2 ± 6.9	40.0 ± 7.6	< 0.001

*EIPH* exercise-induced pulmonary hypertension.

### Association between clinical outcomes and EIPH at peak workload

Of 57 patients with EIPH at peak workload, early surgical intervention was indicated in 38 patients (67%), and the other 19 patients (33%) were observed using the watchful waiting strategy. Conversely, of 66 patients without EIPH, early surgical intervention was indicated in 27 patients (41%), and the other 39 patients (59%) were observed using the watchful waiting strategy. The reasons for early surgical intervention in patients without EIPH were as follows; symptoms evoked by exercise were in 8 patients (30%), new-onset atrial fibrillation during exercise was in 3 patients (11%), and LA dilatation (≥ 60 mL/m^2^) was in 16 patients (59%). Totally, early surgical intervention was performed in 65 patients (53%).

**Figure 1:**
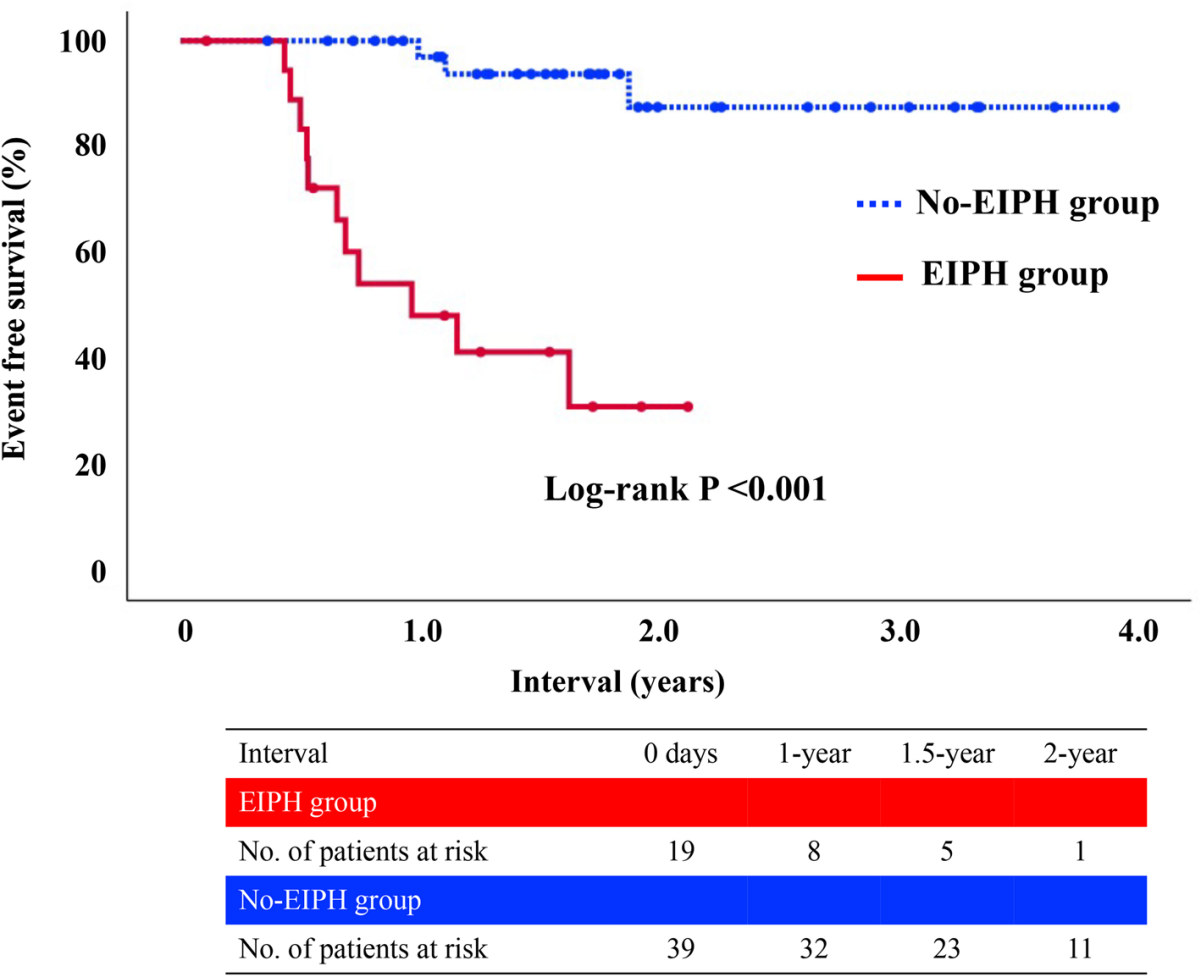
A Kaplan–Meier curve of the cumulative event free survival rates of the endpoint for 2 groups of patients with or without EIPH at peak workload. *EIPH* exercise-induced pulmonary hypertension.

Of 58 patients (47%) with the watchful waiting strategy (EIPH group; 19 patients, no-EIPH group; 39 patients), median follow-up period was 1.5 years (interquartile range: 0.9–1.9). During follow-up, the endpoint occurred in 14 patients [EIPH group; 11 patients (58%), no-EIPH group; 3 patients (8%)]; death in one patient, hospitalization for heart failure in 5 patients, and worsening of symptoms in 8 patients. The event free survival was lower in a EIPH group than in a no-EIPH group (48.1 vs. 97.0% at 1-year, long-rank p < 0.001) (Fig. 1).

### Data of ESE at rest and low workload

The data of ESE at rest and at low workload are presented in Tables 3 and 4. LV and LA size and quantitative evaluation of MR at rest were similar between the EIPH and no-EIPH groups. The values of ejection fraction at rest and TRPG at low workload were higher and eʹ at rest was lower in a EIPH group than in a no-EIPH group (ejection fraction at rest, 64.1 ± 3.5 vs. 62.9 ± 2.4%, p = 0.030; TRPG at low workload, 46.7 ± 10.0 vs. 35.4 ± 9.8 mmHg, p < 0.001; eʹ at rest, 9.0 ± 2.3 vs. 10.1 ± 3.0 cm/s, p = 0.025). Other LV function, LA function, right ventricular function, vital sign, and MR grade at rest and low workload were similar between the 2 groups. There were also no significant differences in the value of TRPG at rest between the 2 groups.

**Table 3:** Hemodynamics and echocardiographic data at rest.

	Overall (N = 123)	EIPH (N = 57)	No-EIPH (N = 66)	p-value
Systolic blood pressure, mmHg	135.8 ± 19.9	137.7 ± 19.0	134.1 ± 20.7	0.33
Diastolic blood pressure, mmHg	82.5 ± 11.9	83.2 ± 10.4	82.0 ± 13.0	0.57
Heart rate, bpm	69.7 ± 12.1	67.8 ± 12.6	71.4 ± 11.4	0.10
Stroke volume, mL	58.0 ± 13.7	59.3 ± 12.8	56.9 ± 14.4	0.33
Cardiac index, L/min/m^2^	2.51 ± 0.68	2.47 ± 0.67	2.54 ± 0.69	0.59
Tricuspid regurgitation pressure gradient, mmHg	23.3 ± 8.6	24.8 ± 8.1	21.9 ± 8.9	0.069
Estimated right atrial pressure, mmHg	3.6 ± 2.0	4.0 ± 2.7	3.3 ± 1.2	0.061
Left ventricular diastolic volume, mL	111.7 ± 34.1	115.3 ± 34.4	108.5 ± 33.8	0.28
Left ventricular systolic volume, mL	40.8 ± 12.7	41.6 ± 12.7	40.0 ± 12.8	0.49
Left ventricular diastolic diameter, mm	53.2 ± 5.7	53.7 ± 4.8	52.8 ± 6.3	0.37
Left ventricular systolic diameter, mm	33.0 ± 4.4	32.9 ± 4.1	33.0 ± 4.6	0.90
Left ventricular ejection fraction, %	63.5 ± 3.0	64.1 ± 3.5	62.9 ± 2.4	0.030
Left ventricular global longitudinal strain, %	− 21.6 ± 3.4	− 21.6 ± 3.5	− 21.7 ± 3.3	0.91
E wave, cm/s	101.9 ± 25.7	103.8 ± 25.0	100.2 ± 26.3	0.44
e′ (average), cm/s	9.6 ± 2.8	9.0 ± 2.3	10.1 ± 3.0	0.025
Left atrial diameter, mm	44.6 ± 11.0	45.2 ± 8.9	44.1 ± 12.6	0.57
Left atrial volume index, mL/m^2^	70.7 ± 41.2	71.3 ± 41.5	70.3 ± 41.4	0.89
Left Atrial reservoir strain, %	28.5 ± 11.3	26.7 ± 11.2	30.1 ± 11.1	0.088
Tricuspid annual plane systolic excursion, mm	23.2 ± 4.4	23.1 ± 3.9	23.3 ± 4.9	0.80
Fractional area change, %	43.9 ± 5.8	44.3 ± 5.5	43.5 ± 6.0	0.49
Right ventricle free wall strain, %	− 24.5 ± 5.6	− 23.9 ± 4.5	− 24.9 ± 6.4	0.33
Severe mitral regurgitation, n (%)	63 (51)	31 (54)	32 (49)	0.51
Mitral regurgitation regurgitant volume, mL	52.1 ± 12.2	53.6 ± 12.0	50.9 ± 12.3	0.23
Mitral regurgitation effective orifice area, cm^2^	0.33 ± 0.10	0.34 ± 0.10	0.32 ± 0.09	0.27

*EIPH* exercise-induced pulmonary hypertension.

**Table 4:** Associated parameters at low workload.

	Overall (N = 123)	Ex PH (N = 57)	No Ex PH (N = 66)	p-value
**At low workload (10 or 25 W)**				
Systolic blood pressure, mmHg	158.1 ± 24.6	161.2 ± 23.3	155.4 ± 25.6	0.20
Diastolic blood pressure, mmHg	90.1 ± 15.1	90.4 ± 14.8	89.8 ± 15.5	0.83
Heart rate, bpm	98.3 ± 13.5	96.9 ± 13.3	99.5 ± 13.7	0.29
Stroke volume, mL	64.7 ± 16.0	66.1 ± 15.7	63.4 ± 16.2	0.35
Cardiac index, L/min/m^2^	3.93 ± 0.98	3.94 ± 1.06	3.92 ± 0.92	0.92
Starting from 10 W, n (%)	9 (7)	4 (7)	5 (8)	0.91
Tricuspid regurgitation pressure gradient, mmHg	40.7 ± 11.4	46.7 ± 10.0	35.4 ± 9.8	< 0.001
Ejection fraction, %	67.2 ± 4.6	67.1 ± 4.5	67.2 ± 4.6	0.87
Left ventricular global longitudinal strain, %	− 24.5 ± 4.3	− 24.1 ± 4.4	− 24.9 ± 4.2	0.34
E wave, cm/s	132.1 ± 28.8	136.2 ± 27.1	128.6 ± 30.0	0.15
e′ (average), cm/s	11.6 ± 3.1	11.3 ± 3.0	11.8 ± 3.2	0.36
Left atrial reservoir strain, %	32.4 ± 14.5	31.1 ± 14.9	33.4 ± 14.1	0.38
Tricuspid annual plane systolic excursion, mm	25.5 ± 5.6	25.1 ± 5.2	25.9 ± 5.9	0.45
Fractional area change, %	45.6 ± 5.6	45.7 ± 4.7	45.5 ± 6.3	0.84
Right ventricle free wall strain, %	− 25.3 ± 6.2	− 24.4 ± 4.7	− 26.1 ± 7.2	0.13
Severe mitral regurgitation, n (%)	72 (59)	36 (63)	36 (55)	0.33

*EIPH* exercise-induced pulmonary hypertension.

### Relationship between TRPG at peak workload and parameters at rest or low workload

**Figure 2:**
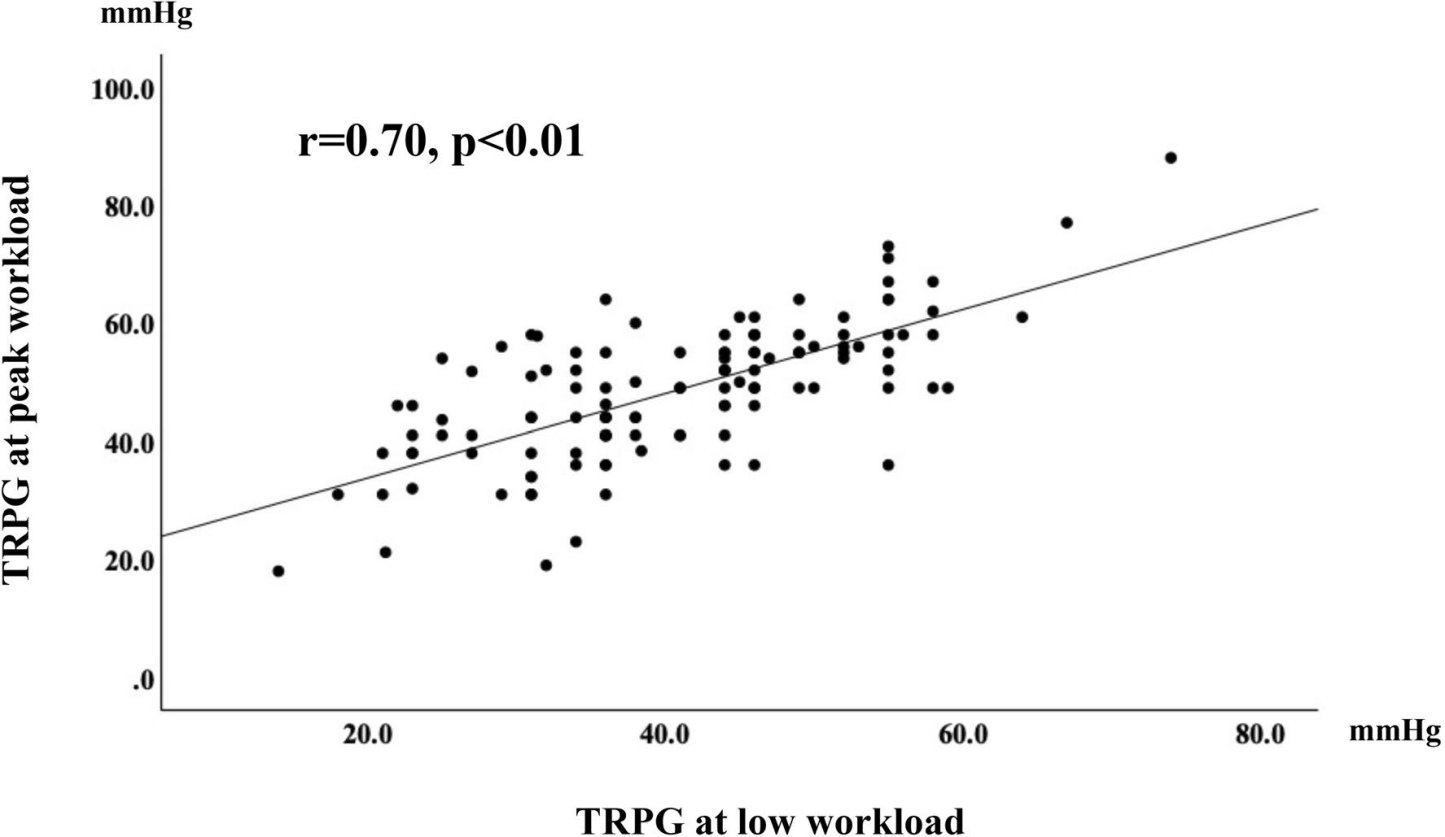
Relationship between TRPG at peak workload and low workload. A significant correlation was found between TRPG at peak workload and TRPG at low workload (r = 0.70, p < 0.001). *TRPG* tricuspid regurgitation peak gradient.

TRPG at peak workload showed a moderate correlation with TRPG at low workload (r = 0.70, p < 0.001) (Fig. 2), although the correlation with age (r = 0.33, p < 0.001), eʹ at rest (r =  − 0.26, p = 0.004), or ejection fraction at rest (r = 0.20, p = 0.027) was weak. In multivariable linear regression analysis, where TRPG at peak workload was set as the dependent variable, TRPG at low workload (β = 0.67, p < 0.001) and eʹ at rest (β =  − 0.15, p = 0.028) were independently associated with TRPG at peak workload, whereas age (β = 0.01, p = 0.87), NYHA II (β = 0.02, p = 0.83), and ejection fraction at rest (β = 0.04, p = 0.55) were not (Table 5).

**Table 5:** Variables associated with TRPG at peak workload.

	Univariable analysis			Multivariable analysis					
	Regression coefficient	β	p-value	Model 1 (R^2^ = 0.49)			Model 2 (R^2^ = 0.50)		
				Regression coefficient	β	p-value	Regression coefficient	β	p-value
Age	0.25	0.33	< 0.001	0.01	0.01	0.94			
NYHA II	3.72	0.17	0.071	0.34	0.02	0.84			
TRPG at low workload	0.71	0.70	< 0.001	0.67	0.66	< 0.001	0.68	0.67	< 0.001
Ejection fraction at rest	0.78	0.20	0.027	0.16	0.04	0.55			
e′ at rest	− 1.10	− 0.26	0.004	− 0.58	− 0.14	0.092	− 0.62	− 0.15	0.028

R^2^ adjusted coefficient of determination.

Model 1 included age, NYHA II, TRPG at low workload, ejection fraction at rest, and e′ at rest.

Model 2 included all variables in Model 1 with stepwise multiple regression analysis.

*NYHA* New York heart association, *TRPG* tricuspid regurgitation pressure gradient.

### Association between prognosis in patients with the watchful waiting strategy and TRPG at peak or low workload

**Figure 3:**
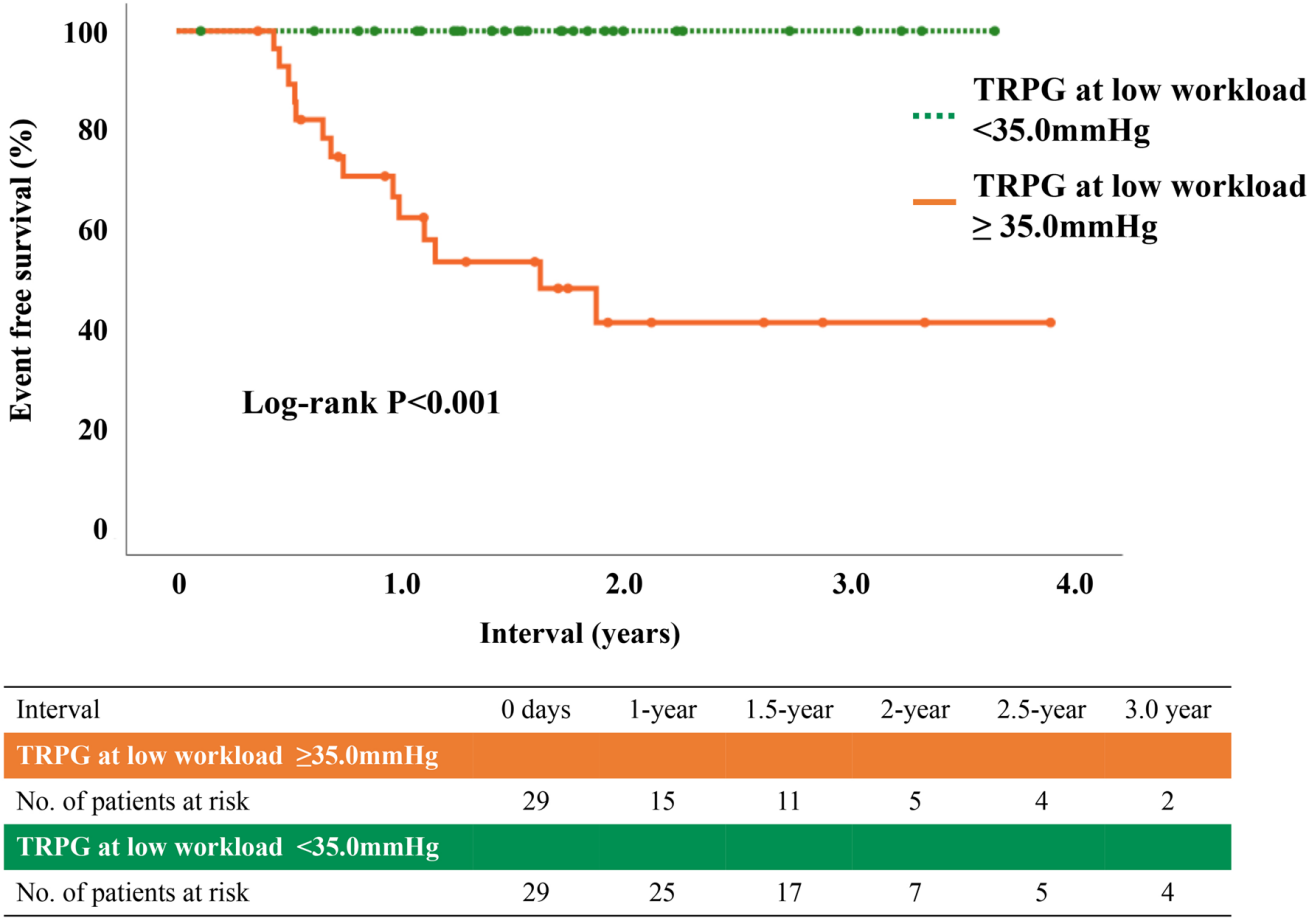
A Kaplan–Meier curve of the cumulative event free survival rates of the endpoint for 2 groups of patients with or without TRPG ≥ 35.0 mmHg at low workload. *TRPG* tricuspid regurgitation peak gradient.

The receiver operating characteristic analysis was performed for predicting prognosis at 2 years after performing ESE in 58 patients with the watchful waiting strategy. The area under the curve based on TRPG at peak workload and TRPG at low workload was 0.89 (95% confidential interval 0.78–0.99) and 0.80 (95% confidential interval, 0.69–0.91), respectively. On the other hand, e′ at rest (AUC of 0.72; 95% confidential interval, 0.57–0.87) did not discriminate prognosis significantly at 2 years after performing ESE in patients the watchful waiting strategy. TRPG at peak workload ≥ 49.5 mmHg predicted prognosis with a sensitivity of 92% and specificity of 81% and the cut-off value was almost same as the definition of EIPH (TRPG 50 mmHg at peak workload). TRPG at low workload ≥ 35.0 mmHg predicted prognosis with a sensitivity of 100% and specificity of 65%. The event free survival was lower in patients with TRPG at low workload ≥ 35.0 mmHg than in patients with TRPG at low workload < 35.0 mmHg (62.4 vs. 100% at 1-year, long-rank p < 0.001) (Fig. 3). In univariate Cox regression analysis, TRPG at low workload [per mmHg; hazard ratio (HR) 1.119; 95% confidence interval (CI) 1.053–1.189; p < 0.001] and TRPG at peak workload (per mmHg; HR, 1.194; 95% CI 1.107–1.289; p < 0.001) were significantly associated with poor prognosis.

## Discussion

The main findings of this study were that (1) prognosis of patients with the watchful waiting strategy and with EIPH was poor compared to patients without EIPH, (2) TRPG at low workload and eʹ at rest were independently associated with TRPG at peak workload; and (3) TRPG at low workload ≥ 35.0 mmHg as well as EIPH were associated with poor prognosis in patients with the watchful waiting strategy.

Symptoms of MR are evaluated using the NYHA classification based on subjective assessment in daily practice, but the reliability and validity of the NYHA classification have been controversial^10,11^. The differences in exercise capacity between NYHA classes I and II are subtle and a significant overlap in 6-min walk distance was noted between the 2 groups^12^. Patients with significant MR with NYHA class II are difficult to be regarded as symptomatic or not, although being symptomatic is a determinant of class I indication for surgical intervention. Therefore, objective evaluation with EIPH by ESE is important to determine the treatment strategy. Previous studies have reported that EIPH developed before emerging symptoms and EIPH predicted the future occurrence of symptoms in asymptomatic patients with severe MR^13,14^. In addition, a few studies have addressed the prognostic relevance of EIPH in valvular heart diseases including MR and aortic stenosis^6,15,16^. However, data about how EIPH affects clinical practice are limited despite the recommendation of ESE for decision-making in the management of asymptomatic or equivocally symptomatic MR in the current gudelines^2–4^. The present study showed that prognosis of patients in a watchful waiting group despite EIPH was poor compared to those without EIPH. Thus, the importance of ESE and evaluating EIPH in asymptomatic patients with severe MR could be re-acknowledged.

In the present study, TRPG at low workload was independently associated with TRPG at peak workload, and elevating TRPG at low workload as well as peak workload predicted poor prognosis in patients with the watchful waiting strategy. In previous studies using cardiopulmonary exercise testing (not ESE), usefulness of low workload exercise has already demonstrated in patients with PH^17,18^. In real-world clinical settings, echocardiographic parameters including TR velocity are difficult to accurately evaluate at peak workload because of poor image under tachycardia and tachypnea. Moreover, assessing TRPG at peak workload is sometimes difficult in elderly patients who cannot achieve sufficient workload. On the other hand, any patients can pedal a bicycle and good images can be easily obtained at low workload. The usefulness of evaluating TRPG at low workload was also supported by the worse prognosis of patients with the watchful waiting strategy and TRPG at low workload ≥ 35.0 mmHg compared to patients with TRPG < 35.0 mmHg. Therefore, evaluating TRPG at low workload instead of TRPG at peak workload using ESE is useful in real-world clinical settings^19^. Other echocardiographic parameters related to MR severity and LV and LA function at rest and low workload were not individually associated with TRPG at peak workload or did not discriminate prognosis significantly (eʹ at rest), although previous studies reported that parameters of LV and LA function during exercise were associated with clinical outcomes^20–23^. MR severity during exercise is another important parameter in stress echocardiography for patients with MR. However, there are several problems in evaluating MR severity during exercise stress test: inaccuracy of qualitative evaluation in patients with an eccentric jet and difficulty in quantitative evaluation of regurgitant volume by PISA method under tachycardia and tachypnea. EIPH is an integrated parameter with individual components of MR severity and LV and LA function. Therefore, EIPH may be a useful parameter in determining treatment strategy and predicting prognosis for asymptomatic MR, and evaluation of EIPH using TRPG at low workload has clinical significance.

There are several limitations in the present study. First, this is a single-center study that included relatively selected patients with preserved LV function, having an ability to exercise, and measuring tricuspid regurgitant velocity during exercise and without concomitant valvular diseases. These findings may not be extrapolated to all patients with MR. Second, although a previous study has shown that exercise effective orifice area is a good predictor of events in patients with MR^5^, we could not measure the quantification of MR at low and peak workload because of technical matter. Finally, we did not perform cardiopulmonary exercise test with measurement of gas exchange, which has the advantage of providing quantitative evaluation of maximal exercise capacity^24^; thus, peak oxygen consumption was not measured as a scale of symptoms.

In conclusion, considering poor prognosis of asymptomatic patients with MR and EIPH, the importance of ESE and evaluating EIPH in patients with MR was re-acknowledged. TRPG at peak workload can be predicted by TRPG at low workload, and TRPG at low workload may be useful in real-world clinical settings.

## Methods

### Study population

**Figure 4:**
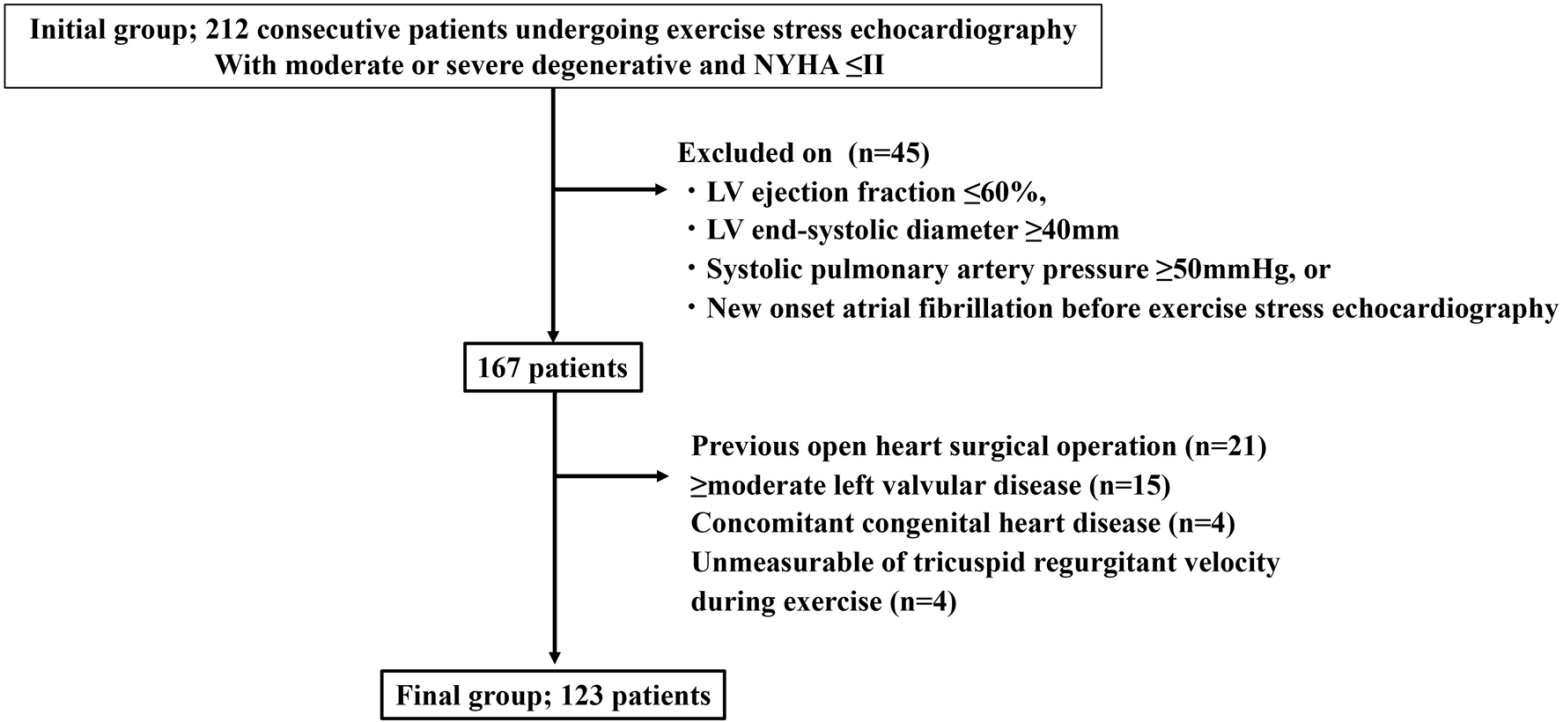
Flowchart of the recruitment of patients. *NYHA* New York heart association, *LV* left ventricular.

We retrospectively enrolled 212 consecutive patients with moderate or severe degenerative MR (effective regurgitant orifice > 20 mm^2^ or regurgitant volume > 30 mL) and New York Heart Association (NYHA) class I or II who were referred for ESE between April 2018 and May 2021. Patients with class I or IIa recommendation for surgical intervention (ejection fraction ≤ 60%, LV end-systolic diameter ≥ 40 mm, systolic pulmonary artery pressure ≥ 50 mmHg at rest, or new-onset atrial fibrillation) (n = 45)^2–4^, previous open-heart surgery (n = 21), ≥ moderate left-sided valvular disease other than MR (n = 15), concomitant congenital heart disease (n = 4), and unmeasurable tricuspid regurgitant velocity during exercise (n = 4) were excluded. Finally, 123 patients were included in the study (Fig. 4). Systematic interview and physical examination were performed by experienced cardiologists and symptomatic status was carefully assessed. Demographic and clinical data were collected at the time of echocardiographic examination. This study was carried out in accordance with the Declaration of Helsinki and approved by the Institutional Research Board of the National Cerebral and Cardiovascular Center, Suita, Japan (R19053-3). All patients provided written informed consent related to perform the ESE and use the data, and the details of the study were disseminated to patients with the opportunity for patients to decline participation in the study (opt-out).

### ESE

Echocardiography was performed using a commercially available system (Vivid 7 and E95, GE Healthcare, Tokyo, Japan). All patients underwent a symptom-limited graded bicycle exercise test in a semi-supine position on a tilting exercise table (Ergometer and Tilt Table 750EC, Lode, Groningen, Netherlands) for continuous 2D echocardiography. Exercise was started at a 3-min workload of 10 or 25 W, which was selected by expert physicians according to age or exercise tolerance. The exercise intensity was increased by 15 W from 10 W and subsequently 25 W every 3 min. Electrocardiograms and vital signs were recorded at each stage. The obtained echocardiographic data at rest, low workload (10 or 25 W), and peak workload were digitally stored and then stored on a workstation for offline analysis (EchoPAC, GE Healthcare, Tokyo, Japan). The degree of dyspnea was evaluated at peak workload by self-assessment according to the original Borg categorical scale and graduated from 6 (no dyspnea) to 20 (maximal imaginable dyspnea). SPAP was obtained from systolic tricuspid regurgitation peak gradient (TRPG) calculated using the modified Bernoulli equation (ΔP = 4v^2^, where v is maximal tricuspid regurgitant jet velocity in m/s) and addition of right atrial pressure estimated by diameter of inferior vena cava and the presence of inspiratory collapse at rest and 10 mmHg for right atrial pressure during exercise as previously described and validated^25^. According to the guidelines, EIPH was defined as SPAP ≥ 60 mmHg or TRPG ≥ 50 mmHg during exercise^2,3,5,6^. The results of ESE were judged by occurrence of symptoms, EIPH, or new-onset atrial fibrillation during exercise^2–4,7^.

### Echocardiographic parameters

Biplane Simpson’s method was used to obtain LV ejection fraction and LA maximal volume. LV stroke volume was calculated by multiplying the LV outflow tract area by the LV outflow tract velocity–time integral measured by pulsed wave Doppler. LV global longitudinal strain was calculated by averaging all segmental strain values from the apical four-chamber, two-chamber, and long-axis views. From an apical four-chamber right ventricle focused view, right ventricle free wall longitudinal strain was calculated by averaging the peak longitudinal strain of the three right ventricle free wall segments, excluding the interventricular septum, to prevent LV interaction. LA dynamics were evaluated using LA strain to assess reservoir function^26^. LA strain was obtained by averaging all segment strain values from the apical four- and two-chamber views. To confirm the accuracy of the echocardiographic parameters, the measurements were repeated by 2 experienced sonographers at our institution being blinded to the clinical outcome.

MR was graded by color flow jet area at rest and exercise as mild (1+), moderate (2+), or severe (3 to 4+) according to the guideline of native valvular regurgitation^27^. MR severity at baseline echocardiography was also quantified using the proximal isovelocity surface area (PISA) method^28,29^.

### Treatment strategy after ESE and prognosis in patients with the watchful waiting strategy

The frequency of undergoing surgical intervention for MR within 6 months after ESE (early surgical intervention) was investigated. Patients who did not undergo surgical intervention within 6 months belonged to a watchful waiting strategy group. The decision-making for surgical intervention was made by the expert physician integrating patient’s wishes, and reasons for surgical indication were collected from medical charts in an early surgical intervention group. In a watchful waiting strategy group, prognosis after ESE was compared between 2 groups divided by pulmonary artery pressure at ESE. The endpoint was a composite of death, hospitalization for heart failure, and worsening of symptoms.

### Statistical analysis

Categorical variables are presented as numbers and percentages and were compared using the χ^2^ test. Continuous variables are expressed as the mean ± standard deviation or median with interquartile range and were compared using Student’s t-test. The event free survival rates of the endpoint in patients with or without EIPH at peak workload and with or without elevating TRPG at peak and low workload were evaluated using Kaplan–Meier analysis and the log-rank test. Cox proportional-hazards analysis was used to evaluate the HR and 95% CI of the parameters associated with the composite endpoint. The correlation between TRPG at peak workload and clinical parameters was investigated using the Pearson correlation coefficient. Univariable and multivariable linear regression analyses with the use of a forward and backward stepwise variable selection procedure were performed to investigate the independent associations between variables. Receiver operating characteristic analyses were performed to investigate the sensitivity and specificity for prognosis at 2 years after ESE and determine the best cutoff value for TRPG at peak and low workload. Statistical analyses were performed using SPSS Statistics for Mac (version 27.0; IBM, Armonk, NY, USA). All reported p-values were two-tailed, and p-values of < 0.05 were considered statistically significant.

## Data Availability

The datasets generated and/or analyzed during the present study are available from the corresponding author upon reasonable request.
